# Supplementary material for: Altered bone growth dynamics prefigure craniosynostosis in a zebrafish model of Saethre-Chotzen syndrome
Source: eLife. 2018 Oct 25;7:e37024. doi: 10.7554/eLife.37024 (PMC6207424; doi:10.7554/eLife.37024)
Supplement: Supplementary file 1. [file elife-37024-supp1.docx]

**Table S1. Summary of phenotypes observed in combinatorial zebrafish mutants**

| **Genotype** | **N** | **Coronal Synostosis** | **Coronal Synostosis Index** | **Sagittal Gaps** | **Ectopic Suture - Metopic** | **Ectopic Suture - Sagittal** |
| --- | --- | --- | --- | --- | --- | --- |
| *12^-/-^*; *1a^+/+^*; *1b^+/+^* | 10 | 0% | 0 | 10% (1/10) | 0% | 10% (1/10) |
| *12^+/-^*; *1a^-/-^*; *1b^+/-^* | 9 | 0% | 0 | 0% | 0% | 22% (2/9) |
| *12^+/+^*; *1a^-/-^*; *1b^-/-^* | 1 | 0% | 0 | 0% | 100% (1/1) | 0% |
| *12^+/-^*; *1a^-/-^*; *1b^-/-^* | 7 | 0% | 0 | 0% | 43% (3/7) | 0% |
| *12^-/-^*; *1a^-/-^*; *1b^-/-^* | 3 | 33% (1/3) | 0.667 | 0% | 33% (1/3) | 0% |
| *12^-/-^*; *1a^+/-^*; *1b^-/-^* | 22 | 41% (9/22) | 1 | 9% (2/22) | 0% | 0% |
| *12^-/-^*; *1a^+/+^*; *1b^-/-^* | 13 | 38% (5/13) | 1.077 | 15% (2/13) | 0% | 0% |

Abbreviations: *12*, *tcf12*; *1a*, *twist1a*; *1b*, *twist1b*.
