# Supplementary material for: Altered bone growth dynamics prefigure craniosynostosis in a zebrafish model of Saethre-Chotzen syndrome
Source: eLife. 2018 Oct 25;7:e37024. doi: 10.7554/eLife.37024 (PMC6207424; doi:10.7554/eLife.37024)
Supplement: Supplementary file 2. [file elife-37024-supp2.docx]

**Table S2. TALEN targeting and mutant genotyping**

| **Gene** | **TALEN Targets** | **Genotyping Primers** | **Annealing Temperature (ºC)** | **Restriction Enzyme** | **Product sizes (bp)** |
| --- | --- | --- | --- | --- | --- |
| *tcf12* | L: TATGGGGGA  ATGCTGGGAGG  R: TGTAGTTTCC  AGACTGTGGC | F: CCACATCTTC  AAAGCTGGAAA  R: TTGATCTGAT  CCCGCAGAG | 58 | BsaBI | WT: 145+99  MT: 244 |
| *twist1a* | L: TGGACAGTCT  GGGAAACAGC  R: TGACGCGCT  TCGGTTGTCGC | F: TGGACAGTCT  GGGAAACAGC  R: GTGGGACTG  TCGGAATCCT | 58 | -- | WT: 109  MT: 102 |
| *twist1b* | L: TCAGCAACA  GCGACGGAGAG  R: TCTTTTCCT  TGCGCACCTTT | F: GCGGACAGT  CTCAGCAACAG  R: TTCTTGCTC  GACCGTCTTTT | 58 | -- | WT: 80  MT: 69 |
